# Bone and Soft Tissue Tumors: New Treatment Approaches

**DOI:** 10.3390/cancers13081832

**Published:** 2021-04-12

**Authors:** Shinji Miwa, Norio Yamamoto, Hiroyuki Tsuchiya

**Affiliations:** Department of Orthopedic Surgery, Graduate School of Medical Science, Kanazawa University, Kanazawa 920-8640, Japan; norinori@med.kanazawa-u.ac.jp (N.Y.); tsuchi@med.kanazawa-u.ac.jp (H.T.)

Bone and soft tissue sarcomas require intensive treatments, including chemotherapy, surgical resection with safe margin, and radiotherapy. Although favorable outcomes have been reported in patients with non-metastatic sarcomas, the outcomes in patients with metastatic or recurrent sarcomas remain unsatisfactory [1]. To overcome the metastatic or recurrent sarcomas, new treatments or modifications of the standard treatment are required. This special issue included recent studies and reviews regarding therapeutic targets, anticancer agents, immunotherapy, and management in patients with bone and soft tissue sarcomas.

This special issue included several studies and reviews of abnormal gene expression in bone and soft tissue sarcomas. Simpson et al. reported that a comparison of gene expression between canine osteosarcomas and non-tumor tissue showed 1281 significantly differentially expressed genes (839 lower and 442 elevated gene expression), a subset of which were validated by qRT-PCR and immunohistochemistry [2]. Furthermore, Greither et al. investigated the influence of miR-155-5p and miR-203a-3p expression on prognosis in patients with soft tissue sarcomas [3]. This study showed that increased expression of miR-155-5p was significantly associated with increased tumor stage, and that high miR-155-5p expression and low miR-203a-3p expression were significantly associated with poor survival in patients with soft tissue sarcomas. In addition, Fellenberg et al. reported the importance of microRNAs as a therapeutic target of osteosarcoma [4]. In this study, silencing of miR-127-3p and miR-376a-3p was identified in osteosarcoma cell lines and tissues, and the proliferation and colony formation of osteosarcoma cells were significantly inhibited by transfection with miR-127-3p and miR-376a-3p mimics. Cells transfected with miR-127-3p and miR-376a-3p showed a significant decrease in tumor volume compared to wildtype cells. These results suggest that these miRNAs are candidate targets for the development of new therapeutic strategies for the treatment of osteosarcoma. Reviews by Czarnecka et al. have comprehensively discussed the gene mutations, molecular biology, therapeutic targets, and recent clinical trials in osteosarcoma and epithelioid sarcoma [5,6]. These sarcomas are known to have a high rate of metastasis and recurrence, and clinical outcomes of patients with metastatic lesions are unsatisfactory. Recent investigations of gene mutations and molecular biology may contribute to the discovery of new therapeutic targets.

Among the various signaling pathways related to tumor progression, the Hippo/YAP signaling pathway is involved in physiological processes and pathologies, such as regulation of tissue regeneration, immunity, stem cell differentiation, and tumors. Morice et al. discussed associations of the Hippo pathway with the progression of pediatric sarcomas in their review article [7]. In their review, the mechanisms of the association of the Hippo/YAP signaling pathway on tumor proliferation, angiogenesis, epithelial-to-mesenchymal transition, migration, and invasion were discussed. Furthermore, several agents targeting the Hippo/YAP pathway have been introduced. However, further investigation of the mechanism and therapeutic targets of the Hippo/YAP pathway in sarcoma cells are required to assess the usefulness of this pathway as a therapeutic target in patients with sarcomas.

Autophagy, which allows the degradation and recycling of cellular components, is upregulated in some cancer stem cells. Camuzard et al. investigated the relationship between cancer stem cells (CSCs) and autophagy in osteosarcoma [8]. They demonstrated that autophagy is more efficient in osteosarcoma CSC-enriched populations than in parental cell lines. Their results suggested that autophagy is an important process for the survival of CSCs in osteosarcoma.

Although the number of available neoadjuvant modalities is continuously growing, the choice of the proper treatment for sarcoma is challenging. Ando et al. investigated the usefulness of a combination of rapamycin and gemcitabine compared with rapamycin and gemcitabine monotherapies [9]. In their study, combination therapy with rapamycin and gemcitabine was more effective than treatment with a single agent in both in vitro and in vivo studies. In a review by Spaleck et al., evidence, indications, and related risks of neoadjuvant treatments for soft tissue sarcomas, including radiotherapy, chemotherapy, targeted therapy, radiosensitizers, hyperthermia, and their combinations, were discussed [10]. Furthermore, current recommendations for neoadjuvant therapy for soft tissue sarcomas are summarized in the review. In a review article by Nakano, the need for precision medicine in soft tissue sarcomas was discussed [11]. Although anticancer agents have been approved according to the tumor subtypes, soft tissue sarcomas have a variety of gene mutations. Although soft tissue sarcomas account for only 1% of malignancies, soft tissue sarcomas have been classified into 50 subtypes according to their clinical and histological features. The rarity and diversity of soft tissue sarcomas makes it difficult to investigate the efficacy and safety of anticancer drugs in clinical trials. Recently, genomic profiling has been widely used in soft tissue sarcomas. Knowledge of the connection between genomic profiling data and optimal treatment strategies is required to determine the optimal clinical treatment strategies for patients with soft tissue sarcomas.

Chondrosarcomas are commonly resistant to chemotherapy and radiation therapy, resulting in limited treatment options. Although the majority of patients with advanced chondrosarcomas are treated with chemotherapy, the number of patients with objective responses to chemotherapy is limited. In this special issue, Monga et al. reviewed recent clinical trials in patients with advanced chondrosarcomas [12]. In contrast, Venneker et al. investigated the influence of poly (ADP-ribose) polymerase (PARP) on response to chemo- and radio-therapy in chondrosarcoma cell lines [13]. In this study, the PARP inhibitor talazoparib slightly induced apoptosis and cell cycle arrest in chondrosarcoma cell lines, and talazoparib sensitized chondrosarcoma cell lines to temozolomide and radiation therapy. This therapeutic approach may improve the sensitivity to chemotherapy and radiation therapy for chondrosarcomas.

Patients with rhabdomyosarcoma require multimodality treatment, including surgical tumor excision, chemotherapy, and radiation therapy. We report a review of current and promising treatments for rhabdomyosarcoma [14]. Although no significant modification of the standard treatment was seen in recent studies, recent clinical trials of anticancer agents, molecular targeted drugs, and immunotherapy have reported the efficacy and safety of this treatment in patients with rhabdomyosarcoma. Furthermore, the latest basic studies on rhabdomyosarcoma are introduced in this review article.

Several clinical studies have demonstrated the benefits of immunotherapies compared to standard systemic treatments, and novel developments have been observed in basic and clinical studies of immunotherapy. Monga et al. retrospectively investigated the efficacy and safety of immune checkpoint inhibitors in patients with soft tissue sarcomas [15]. Eighty-eight patients underwent treatment with pembrolizumab, nivolumab, ipilimumab, or a combination of the agents. Among the study patients, 32% of undifferentiated pleomorphic sarcoma (UPS) and 46% of leiomyosarcomas showed objective responses to the treatment. Based on these data, immune checkpoint inhibitors may be candidates for the treatment of patients with UPS and leiomyosarcoma.

This special issue included articles regarding the management and assessment of functional outcomes and quality of life in patients with bone and soft tissue tumors. Smolle et al. investigated correlations between various risk factors and oncological outcomes in 3016 patients with high-grade soft tissue sarcomas, and developed two flexible parametric competing risk regression models for local recurrence and metastasis to support individual follow-up in patients with soft tissue sarcomas [16]. The risk regression model may enable the individualized prediction of local recurrence and distant metastasis in each patient. Saebye et al. evaluated the development of functional outcomes and quality of life within the first year after tumor excision [17]. Although a significant reduction in limb function was observed after tumor excision, improvement in functional outcomes was observed during the first year after the surgery. Soomers et al. investigated the association of time to diagnosis with health-related quality of life (HRQoL) in sarcoma survivors [18]. In this study, 1099 sarcoma patients completed a questionnaire on HRQoL, the time to diagnosis, the perceived impact of the diagnostic interval on HRQoL, and coping. The perceived impact of the diagnostic interval length was associated with the HRQoL of sarcoma survivors, whereas the actual length was not associated with HRQoL.

Patients with postoperative wound complications require additional surgery, prolonged use of antibiotics, and postpone of postoperative chemotherapy or radiation therapy. Therefore, it is thought that postoperative wound complications may exacerbate the clinical outcomes in patients with bone and soft tissue tumors. Dadras et al. investigated the relationship between postoperative wound complications and oncological outcomes in 102 patients with soft tissue sarcomas of the chest wall [19]. Among the study patients, 11 (11%) experienced wound complications after tumor excision. The study showed that cardiovascular morbidity and operation time were significantly correlated with wound complications, and that patients with wound complications had significantly worse local recurrence-free survival and disease-specific survival. These results suggest that surgeons are required to assess the risk of wound complications when determining the surgical procedure in patients with soft tissue sarcomas of the chest walls.

The bone tumor DUX (Bt-DUX) questionnaire is a useful tool for measuring quality of life in patients with bone tumors, which is easy to use in clinical practice. Morri et al. translated the English bone tumor DUX for patients with lower extremity bone tumors into Italian, and evaluated the validity of the Italian version of Bt-DUX [20]. In their study, the Italian version showed a similar usefulness to the Dutch and English versions. The availability of this scale in different languages is important for facilitating international and multicenter studies.

In summary, this special issue of *Cancers* is a collection of articles discussing the latest basic and clinical research of bone and soft tissue tumors. Since bone and soft tissue tumors are rare tumors, it is more difficult to conduct research that leads to new treatments compared to other tumors. However, recent basic and clinical studies have raised candidates for new therapeutic targets, which may improve clinical outcomes in patients with bone and soft tissue tumors. Furthermore, reports of this special issue may contribute to improvements in the management of bone and soft tissue sarcomas. We greatly appreciate all of the authors who contributed to the studies published in this special issue.

## References

[1] Bielack S.S., Kempf-Bielack B., Delling G., Exner G.U., Flege S., Helmke K., Kotz R., Salzer-Kuntschik M., Werner M., Winkelmann W. (2002). Prognostic factors in high-grade osteosarcoma of the extremities or trunk: An analysis of 1702 patients treated on neoadjuvant cooperative osteosarcoma study group protocols. J. Clin. Oncol..

[2] Simpson S., Dunning M., de Brot S., Alibhai A., Bailey C., Woodcock C.L., Mestas M., Akhtar S., Jeyapalan J.N., Lothion-Roy J. (2020). Molecular characterisation of canine osteosarcoma in high risk breeds. Cancers.

[3] Greither T., Koser F., Holzhausen H.J., Guttler A., Wurl P., Kappler M., Wach S., Taubert H. (2020). MiR-155-5p and MiR-203a-3p are prognostic factors in soft tissue sarcoma. Cancers.

[4] Fellenberg J., Lehner B., Saehr H., Schenker A., Kunz P. (2019). Tumor suppressor function of miR-127-3p and miR-376a-3p in osteosarcoma cells. Cancers.

[5] Czarnecka A.M., Synoradzki K., Firlej W., Bartnik E., Sobczuk P., Fiedorowicz M., Grieb P., Rutkowski P. (2020). Molecular biology of osteosarcoma. Cancers.

[6] Czarnecka A.M., Sobczuk P., Kostrzanowski M., Spalek M., Chojnacka M., Szumera-Cieckiewicz A., Rutkowski P. (2020). Epithelioid sarcoma-from genetics to clinical practice. Cancers.

[7] Morice S., Danieau G., Redini F., Brounais-Le-Royer B., Verrecchia F. (2020). Hippo/YAP Signaling pathway: A promising therapeutic target in bone paediatric cancers?. Cancers.

[8] Camuzard O., Trojani M.C., Santucci-Darmanin S., Pagnotta S., Breuil V., Carle G.F., Pierrefite-Carle V. (2020). Autophagy in osteosarcoma cancer stem cells is critical process which can be targeted by the antipsychotic drug thioridazine. Cancers.

[9] Ando T., Ichikawa J., Fujimaki T., Taniguchi N., Takayama Y., Haro H. (2020). Gemcitabine and rapamycin exhibit additive effect against osteosarcoma by targeting autophagy and apoptosis. Cancers.

[10] Spalek M.J., Kozak K., Czarnecka A.M., Bartnik E., Borkowska A., Rutkowski P. (2020). Neoadjuvant treatment options in soft tissue sarcomas. Cancers.

[11] Nakano K., Takahashi S. (2020). Precision medicine in soft tissue sarcoma treatment. Cancers.

[12] Monga V., Mani H., Hirbe A., Milhem M. (2020). Non-conventional treatments for conventional chondrosarcoma. Cancers.

[13] Venneker S., Kruisselbrink A.B., Briaire-de Bruijn I.H., de Jong Y., van Wijnen A.J., Danen E.H.J., Bovee J. (2019). Inhibition of PARP sensitizes chondrosarcoma cell lines to chemo- and radiotherapy irrespective of the IDH1 or IDH2 mutation status. Cancers.

[14] Miwa S., Yamamoto N., Hayashi K., Takeuchi A., Igarashi K., Tsuchiya H. (2020). Recent advances and challenges in the treatment of rhabdomyosarcoma. Cancers.

[15] Monga V., Skubitz K.M., Maliske S., Mott S.L., Dietz H., Hirbe A.C., Van Tine B.A., Oppelt P., Okuno S., Robinson S. (2020). A retrospective analysis of the efficacy of immunotherapy in metastatic soft-tissue sarcomas. Cancers.

[16] Smolle M.A., Sande M.V., Callegaro D., Wunder J., Hayes A., Leitner L., Bergovec M., Tunn P.U., van Praag V., Fiocco M. (2019). Individualizing follow-up strategies in high-grade soft tissue sarcoma with flexible parametric competing risk regression models. Cancers.

[17] Saebye C., Amidi A., Keller J., Andersen H., Baad-Hansen T. (2020). Changes in functional outcome and quality of life in soft tissue sarcoma patients within the first year after surgery: A prospective observational study. Cancers.

[18] Soomers V., Desar I.M.E., van de Poll-Franse L.V., van de Sande M.A.J., de Haan J.J., Verhoef C., Vriens I.J.H., van Houdt W.J., Bonenkamp J.J., van der Graaf W.T.A. (2020). The perceived impact of length of the diagnostic pathway is associated with health-related quality of life of sarcoma survivors: Results from the dutch nationwide SURVSARC study. Cancers.

[19] Dadras M., Koepp P., Wagner J.M., Wallner C., Sacher M., Lehnhardt M., Behr B., Harati K. (2019). Negative impact of wound complications on oncologic outcome of soft tissue sarcomas of the chest wall. Cancers.

[20] Morri M., Bekkering P.W., Cotti M., Meneghini M., Venturini E., Longhi A., Mariani E., Forni C. (2020). Cross-cultural validation of the italian version of the Bt-DUX: A subjective measure of health-related quality of life in patients who underwent surgery for lower extremity malignant bone tumour. Cancers.

